# Case report: anaplastic lymphoma kinase (*ALK*) rearranged adenocarcinoma with high level of microsatellite instability response to pembrolizumab

**DOI:** 10.3389/fonc.2023.1110638

**Published:** 2023-04-11

**Authors:** Naoko Shigeta, Shuji Murakami, Tomoyuki Yokose, Yohei Miyagi, Haruhiro Saito

**Affiliations:** ^1^ Department of Thoracic Oncology, Kanagawa Cancer Center, Yokohama, Japan; ^2^ Department of Pathology, Kanagawa Cancer Center, Yokohama, Japan; ^3^ Molecular Pathology and Genetics Division, Kanagawa Cancer Center Research Institute, Yokohama, Japan

**Keywords:** non-small cell lung cancer, anaplastic lymphoma kinase rearrangement, immune checkpoint inhibitor, microsatellite instability, case report, programmed death ligand (PD-L) 1, adenocarcinoma, advanced lung adenocarcinoma

## Abstract

The presence of anaplastic lymphoma kinase (*ALK)* rearrangement is reported to be related to the lack of efficacy of immune checkpoint inhibitors (ICIs). High levels of microsatellite instability (MSI-high) are important biomarkers of ICIs, particularly in colorectal cancer. The therapeutic effect of ICIs for MSI-high NSCLC is uncertain because of the rarity of these tumors. Here we report a case of *ALK* rearranged NSCLC with MSI-high. A 48-year-old male was diagnosed with lung adenocarcinoma, cT4N3M1a, stage IVA with *ALK* rearrangement, high PD-L1 expression with a tumor proportion score (TPS) of 100%, and MSI-high. The patient was treated with alectinib as the first-line therapy but progressed at five months with left atrial invasion re-expansion. The patient discontinued alectinib and was switched to pembrolizumab monotherapy. After two months, left atrial invasion significantly decreased. The patient continued pembrolizumab for a year without noticeable adverse events, and tumor shrinkage persisted. This case supports the efficacy of ICIs for MSI-high NSCLC, even in the presence of *ALK* rearrangement.

## 1 Introduction

Anaplastic lymphoma kinase (*ALK*) rearrangement has been detected in 3–5% of non-small cell lung cancers (NSCLC). ALK inhibitors are effective and are considered the standard initial therapy for patients with *ALK*-positive NSCLCs. Unfortunately, despite the initial clinical benefit, acquired resistance to alectinib usually develops in all patients with a median progression-free survival (PFS) of approximately 2–3 years (1). If the resistance mechanism is based on “on-target,” switching to other ALK tyrosine kinase inhibitors (TKIs) has been reported to be effective however, if it is based on “off-target,” systemic therapy similar to the treatment for driver mutation-negative advanced NSCLC is recommended. However, some retrospective analyses have reported a lack of efficacy of immune checkpoint inhibitors (ICIs) in patients with *ALK*-positive NSCLC (2).

A high level of microsatellite instability (MSI-high) is the cause of numerous mutation accumulations at microsatellites, which are short sequence stretches spread over the entire genome. As a result of deficiencies in the DNA mismatch repair system (dMMR), errors during DNA replication, such as insertions or deletions, are not corrected. Thus, MSI is closely associated with cancer development. Patients with MSI-high solid tumors were likely to respond to ICI monotherapy. Pembrolizumab is approved for colorectal cancer with MSI-high or positive for immunohistochemistry of dMMR. However, the therapeutic effect of ICIs on MSI-high NSCLC is still unclear because of the rarity of this cancer. Moreover, the treatment of *ALK*-positive NSCLC with high MSI has not been established. Herein, we present a case of advanced MSI-high and *ALK*-positive lung adenocarcinoma. The patient had an enduring response to pembrolizumab and achieved 1-year progression-free survival (PFS).

## 2 Case presentation

**Figure 1:**
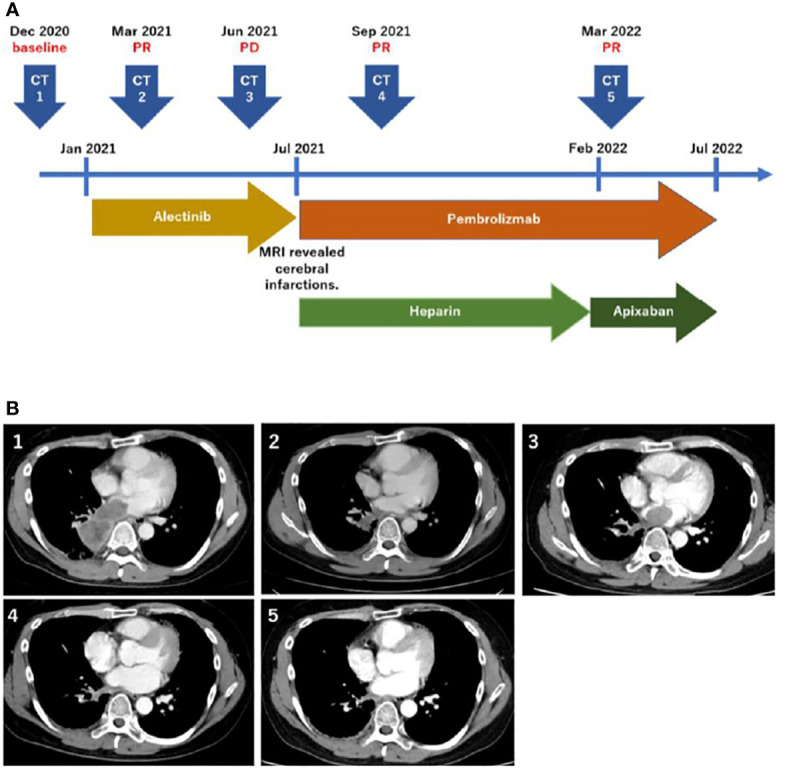
Timeline of the treatment and disease course **(A)**. Computed tomography scans corresponding to the timeline **(B)**.

**Figure 2:**
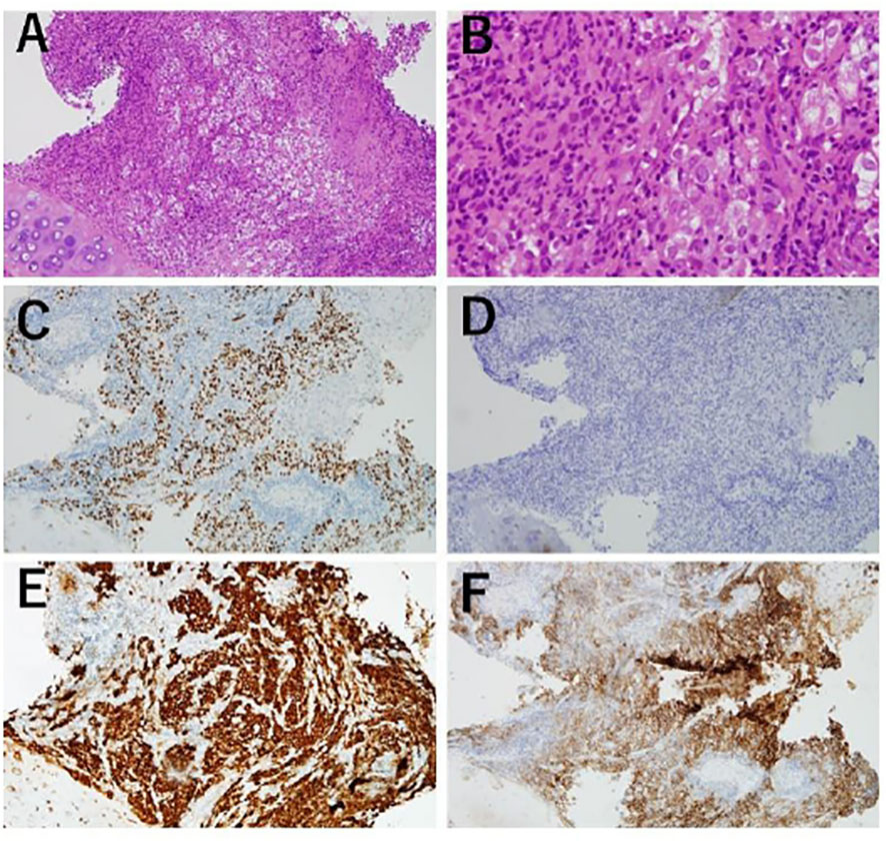
Transbronchiallung biopsy before treatment. **(A, B)** Atypical cell infiltration and lymphocyte infiltration were observed. (100x, 400x) **(C)** Tumor cells positive by immunohistochemistry (IHC) for TTF-1. (100x) **(D)** Tumor cells were negative by IHC for p40. (100x) **(E)** Tumor cells positive for ALK IHC (Roche-Ventana, D5F3). (100x) **(F)** PD-L1 IHC (Dako, 22C3), giving a tumor proportion score of 100. (100).

**Figure 3:**
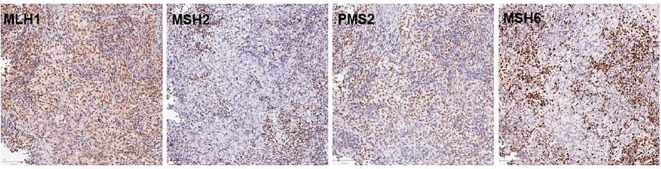
Immunohistochemistry for MMR proteins MLH1, MSH2, PSM2, and MSH6. The nuclei of tumor cell were clearly positive for MLH1 and PMS2 staining together with the surrounding non-neoplastic cell nuclei, largely of infiltrating lymphocytes. In contrast. MSH2 and MSH6 were negative in tumor cells with positive staining for non-neoplastic cell nuclei, serving as internal positive control for staining. The bars in the lower left corner indicate 100mm.

A 48-year-old male patient was admitted to our institution with a primary complaint of a cough and back pain. The patient had a smoking history of 14 pack-years, and no other medical history or cancer. Chest computed tomography (CT) revealed a mass with a diameter of 77 mm in the lower lobe of the right lung with left atrial invasion (**Figure 1B** CT1), enlarged mediastinal lymph nodes (#2R, 4R, #7, and # 2L), right hilar lymph nodes, and right pleural effusion (**Figure 1A**). Transbronchial lung biopsy histopathological immunohistochemistry (IHC) revealed TTF-1 positive adenocarcinoma (**Figures 2A–D**). Based on these results, the patient was diagnosed with lung adenocarcinoma, cT4N3M1a, stage IVA. Additionally, the IHC analysis indicated that the tumor cells were positive for ALK antibody (clone D5F3, VENTANA). (**Figure 2E**) Molecular analysis using the Oncomine Dx Target Test Multi-CDx System (Life Technologies corporation, Frederic and Pleasanton facility, USA) and Oncomine Comprehensive Assay v3 (OCA v3; Thermo Fisher Scientific) using fresh-frozen tissues, which was performed in a lung cancer genomic screening project for individualized medicine in Japan (LC-SCRUM)revealed *an EML4-ALK* fusion V1 (E13:A20). The IHC analysis using programmed death-ligand 1 (PD-L1) IHC 22C3 pharmDx assay showed high PD-L1 expression with a tumor proportion score (TPS) of 100%. (**Figure 2F**) Additionally, analysis by electrophoresis of the PCR products revealed that the case was MSI-high. We performed immunohistochemistry for mismatch repair proteins (MMR) MLH1, MSH2, MSH6, and PMS2. The nuclei of the tumor cells were positive for MLH1 and PMS2 but negative for MSH2 and MSH6 indicating MSI status with MSH2 gene disruption (**Figure 3**).

The patient was treated with alectinib at the Japanese-approved dose of 300 mg twice a day. The tumor size decreased two months after the initiation of alectinib therapy (**Figure 1B** CT2), and he continued its intake as prescribed without any adverse events. However, five months after alectinib treatment, the patient experienced pain in the left fingertips and was diagnosed with peripheral arterial occlusive disease. Brain magnetic resonance imaging (MRI) revealed multiple cerebral infarctions, and laboratory tests demonstrated elevated D-dimer (21.6 μg/ml) levels. Additionally, the CT scan showed left atrial invasion progression (**Figure 1B** CT3). The patient was then put on Heparin therapy for the treatment of diagnosed cancer-associated thromboembolism. Subsequently, alectinib was discontinued and the patient was switched to pembrolizumab IV therapy at 200 mg every three weeks. After evidence of ALK inhibitor resistance, the combined therapy with cytotoxic chemotherapy and pembrolizumab is one of the preferred treatments, regardless of PD-L1 expression. However, because this patient exhibited MSI-high, pembrolizumab monotherapy can be considered as an alternative. After discussing with the patient, he decided to receive pembrolizumab monotherapy. The left atrial invasion was significantly decreased two months after pembrolizumab therapy was initiated (**Figure 1B** CT4). Subsequently, D-dimer levels also returned to normal levels. The patient continued pembrolizumab therapy for a year with no adverse events, and continued tumor shrinkage (**Figure 1B** CT5). Heparin was switched to Apixaban, which the patient continues to use (**Figure 1A**).

## Discussion

Alectinib, a highly selective inhibitor of ALK, is one of the recommended first-line treatments for ALK-positive NSCLC. The median PFS with first-line alectinib was 34.8 months in the ALEX study, however, some patients acquired resistance to alectinib earlier (1). High expression of PD-L1 and smoking history are associated with acquisition of resistance. A retrospective study for ALK-positive NSCLC patients who received second-generation ALK-TKIs including alectinib, showed that median PFS was shorter in patients with high PD-L1 expression (median PFS in patients with PD-L1 TPS of 0% vs 1–49% vs ≥ 50% were 27.43 months vs 30.63 months vs 9.50 months, respectively, P = 0.001) (3). In this case, early tumor progression at only six months was observed despite the initial tumor shrinkage with alectinib, which was consistent with previous reports of poor response to alectinib, as the patient was a smoker and had high PD-L1 expression.

For patients with driver-negative NSCLC with high PD-L1 expression (≥50%), ICIs with or without cytotoxic chemotherapy is recommended. However, ICIs are generally less efficacious in patients with mutations such as *EGFR*, *ALK*, and *ROS-1* (4). The efficacy of ICIs in patients with *EGFR* -positive NSCLC with high PD-L1 expression has been reported, however the efficacy of this group of drug for patients with *ALK* rearrangement is unknown (2, 5). S. Baldacci et al. reported the first case of *ALK*-positive NSCLC with complete and prolonged response to ICI monotherapy (6). In this study, the patient’s first-line treatment was ceritinib, an ALK-TKI, lasted for only five months with tumor progression. Subsequently, two months after initiation of nivolumab therapy, a complete response was observed. Early resistance to ALK-TKIs and the efficacy of ICIs were similar to those in our case. The case reported by Baldacci et al. also showed high PD-L1 expression (100%), which could be a reason for these common therapeutic effects. This case and ours raise hopes for the potential use of ICIs in patients with *ALK*-positive NSCLC having high PD-L1 expression.

In addition to high PD-L1 expression, the present case showed MSI-high. To the best of our knowledge, there are no data on ICIs in patients with MSI-high NSCLC harboring driver mutation. Our case report potentially provides information about a better medication for these patients. These ICIs are effective in patients with MSI-high colorectal cancer and other MSI-high solid tumors, however, the therapeutic effect of MSI-high NSCLC is unclear due its low frequency. The frequency of MSI-high in NSCLC is reported to be only 0.19 – 2.0% (7–9). We performed immunohistochemistry, uses anti-human antibody clones against MSH6, PMS2, MLH1 and MSH2, for mismatch repair proteins (MMR). This case was negative for MSH2 and MSH6. M.E. Salem et al. examined 1057 MSI-high tumors and reported MMR mutations occur in less than half of MSI-high tumors. They found loss of MSH2/MSH6 co-expression is associated with a higher tumor mutation burden compared to loss of MLH1/PMS2 (10). The one limitation of this case is that the patient was not tested for Lynch syndrome, which is caused by germline alterations in MMR genes. Nevertheless, the possibility of Lynch syndrome-associated NSCLC was low because NSCLC is not one of the recognized Lynch syndrome-associated tumors and he had no other malignancy.

Notably, MSI-high NSCLC often has some mutations, which are reported in 68 – 100% of cases (7, 8). Warth et al. analyzed 480 cases of lung adenocarcinoma and found that 4 of them had high MSI, and all of them had mutations in *EGFR* (n=2), *KRAS* (n=1), or *BRAF* (n=1) (8). A cohort of 526 Brazilian patients with NSCLC found only one case of high MSI with *TP53* mutations (7). In 12485 Chinese lung cancer cases, 67 were MSI-high. Of these, 46 had mutations, most of which were *EGFR* (n=26) and the others were *KRAS* (n=5), *RET* (n=2), *PIK3CA* (n=8), *and FGFR2/3* (n=5) (9). These results suggest that driver mutations are frequent in cases with high MSI. This case report provides new insights into the treatment of MSI-high NSCLC with driver mutations.

## Conclusion

We have described a case of advanced lung adenocarcinoma with *ALK* rearrangement, high PD-L1 expression, and MSI-high, in which shrinkage persisted for a year with patient on pembrolizumab therapy. This case supports the efficacy of ICI for MSI-high lung cancer, even in the presence of ALK rearrangement.

## Data availability statement

The original contributions presented in the study are included in the article/supplementary materials. Further inquiries can be directed to the corresponding author.

## Ethics statement

Ethical review and approval was not required for the study on human participants in accordance with the local legislation and institutional requirements. The patients/participants provided their written informed consent to participate in this study. Written informed consent was obtained from the individual(s) for the publication of any potentially identifiable images or data included in this article.

## Author contributions

SM and NS collected the clinical information, diagnostic information, therapeutic information, and images of the patients. NS wrote the manuscript. SM wrote some sections of manuscript. TY and NS collected the pathological images of the patients. YM performed immunohistochemistry for mismatch repair proteins. SM, TY, HS revised the manuscript. All authors contributed to the article and approved the submitted version.
